# Urotensin-ⅡReceptor Antagonist SB-710411 Protects Rat Heart against Ischemia-Reperfusion Injury via RhoA/ROCK Pathway

**DOI:** 10.1371/journal.pone.0146094

**Published:** 2016-01-15

**Authors:** Sheng-Yong Luo, Shuo Chen, Yi-De Qin, Zhi-Wu Chen

**Affiliations:** 1 Department of Pharmacology, Anhui Medical University, Hefei, Anhui, China; 2 Anhui academy of medical sciences, Hefei, Anhui, China; 3 Xinglin College of Liaoning University of Traditional Chinese Medicine, Shenyang, Liaoning, China; 4 Department of Biochemistry and Molecular Biology, Anhui Medical University, Hefei, Anhui, China; University of Pecs Medical School, HUNGARY

## Abstract

**Aim:**

SB-710411 is a rat selective urotensin-II (U-II) receptor antagonist, which can block U-II-induced contraction of the aorta and inhibit U-II-induced myocardial fibrosis in rats. However, the effect of SB-710411 on myocardial ischemia-reperfusion (I/R) injury is unclear. The present study was designed to investigate whether SB-710411 has a protective effect on myocardial I/R injury in rats and the possible mechanisms.

**Methods and Results:**

Myocardial I/R injury was induced by occluding the left anterior descending coronary artery in adult male Sprague-Dawley rats. Hemodynamic parameters, electrocardiogram (ECG), infarct size, histological alteration, lactate dehydrogenase (LDH), creatine phosphokinase-MB (CK-MB), cardiac troponin I (cTnI), RhoA, and the protein expressions of U-II receptor (UTR), ROCK_1_ and ROCK_2_ were evaluated. Cardiac I/R injury significantly up-regulated the expressions of UTR, ROCK_1_ and ROCK_2_ proteins in rat myocardium. SB-710411 1.0 and 2.0 μg/kg significantly reduced cardiac I/R-induced the infarct size and histological damage in rat myocardium, markedly inhibited the changes of hemodynamic parameters and the increases of ST-segment in ECG, the serum LDH and CK-MB activities and cTnI level in rats subjected to myocardial I/R injury. Furthermore, SB-710411 obviously prevented myocardial I/R-increased RhoA activity and UTR, ROCK_1_ and ROCK_2_ protein expressions.

**Conclusions:**

Our results indicate that cardiac I/R injury increases myocardial UTR expression, and SB-710411 has a potent protective effect on myocardial I/R injury in rats. The cardioprotection may be associated with the inhibition of UTR-RhoA/ROCK pathway.

## Introduction

Ischemic heart disease remains one of the primary causes of mortality and morbidity worldwide. Its global prevalence is increasing continuously [1–2]. A pivotal step in avoiding the heart damage is to recover the heart blood supply immediately. However, abrupt reperfusion of ischemic myocardium can augment the injury of heart structure and function. This phenomenon is termed as ischemia-reperfusion (I/R) injury. There are some agents or methods used to protect myocardial I/R injury, such as insulin, bradykinin, atorvastatin, microRNA-320, cyclosporine, ischemia preconditioning, ischemia postconditiong and so on [3]. However, the clinical applicability of them is neither assured nor practical. Thus, elucidating mechanisms of myocardial I/R injury and finding new agents to protect the heart from the damage are of considerable importance.

Urotensin-II (U-II) was firstly isolated from the teleost fish in 1980 [4]. Subsequent studies have shown that U-II occurs in all mammals and acts as a vasoactive peptide. Human U-II is cyclic neuropeptide composed of 11 amino acids and activates the orphan G protein-coupled receptor 14, currently renamed U-II receptor (UTR) to exert various biological effects including regulation of behaviors, neuroendocrine activities and control of cardiovascular functions. Human U-II has been characterized as the most forceful arterial vasoconstrictors with 10–100 times more potent than endothelin-1 [5]. Large amount of data has demonstrated that the concentration of plasma human U-II is elevated in the cardiovascular diseases such as heart failure [6], atherosclerosis [7], hypertension [8–9], ischemia [10], chronic hypoxia [11]. Moreover, serum human U-II level was positively correlated with the degree of myocardium injury in a rat myocardial infarction model. Up-regulation of UTR may stimulate vascular smooth muscle proliferation [12], worsen cardiac hypertrophy [13] and myocardial I/R injury [14], increase cardiac fibrosis and collagen synthesis, and decrease coronary blood flow. In the isolated rat hearts, U-II caused myocardial injury under normal perfusion, and worsened I/R injury, in which UTR were up-regulated [15]. However, the beneficial effects of human U-II on the cardiovascular system have also been reported, it was found that human U-II can alleviate myocardial reperfusion injury via increasing the coronary blood flow and reducing cardiac contractility [16]. Human U-II also has a cardioprotective effect on I/R injury of heart through antioxidant pathway. Therefore, roles of U-II/UTR on cardiovascular pathological process are still conflicting, and need to be clearly ascertained.

SB-710411 (Cpa-D-Cys-Pal-D-Trp-Lys-Val-Cys-Cpa-NH2) is one of UTR antagonists, which can block human U-II-induced contraction in rat isolated aorta and inhibit human U-II-induced myocardial fibrosis [17–18]. However, the effect of SB-710411against myocardial I/R injury has not been reported. It is well known that RhoA/Rho-kinase (Rho-associated coiled-coil-containing protein kinase, ROCK) signaling pathway plays an important role in a variety of cardiovascular diseases [19]. Blocking this pathway can reduce the vascular tone and protect myocardial I/R injury [20–21]. In addition, some studies have shown that RhoA/ROCK pathway is involved in the human U-II-induced physiological and pathological processes such as heart contraction, arterial smooth muscle cell proliferation, cardiac diastolic dysfunction and so on [22]. The present study was therefore designed to investigate the protective effect of SB-710411 on myocardial I/R injury in rats and possible role of RhoA/ROCK pathway in the protection with further exploration of the underlying mechanism.

## Materials and Methods

### Reagents

SB-710411 was custom synthesize by GL Biochem Ltd. (Shanghai, China). Verapamil was purchased from Shanghai Harvest Pharmaceutical Co. Ltd. (Shanghai, China). G-LISA RhoA activation assay biochemistry kit was purchased from Cytoskeleton (Denver, USA). UTR antibody (1:200) was purchased from WuXi Apptec (San Diego, USA), Mouse monoclonal antibodies against ROCK_1_ and ROCK_2_ (1:1000) were purchased from Nanjing Enogene Biological Co (Nanjing, China). 2,3,5-triphenyltetrazolium chloride (TTC) and Evans Blue were purchased from Sigma-Aldrich (St. Louis, Mo., USA). Lactate dehydrogenase (LDH) and Creatine phosphokinase-MB (CK-MB) were purchased from Nanjing Jiancheng Institute of Biotechnology (Nangjing, China). Cardiac troponin I (cTnI) enzyme-linked immunosorbent assay (ELISA) test kit was purchased from Suzhou Calvin Biotechnology Co., Ltd (Suzhou, China).

### Animals

Male Sprague-Dawley rats weighing 250–300g were obtained from the Anhui Medical University Animal Center. This study was carried out in strict accordance with the recommendations in the Guide for the Care and Use of Laboratory Animals of the National Institutes of Health (NIH Publication, 8th edition, 2011). The protocol was approved by the Committee on the Ethics of Animal Experiments of Anhui Medical University.

### Experimental protocol

Sprague-Dawley rats were randomly divided into the following 6 groups: sham, control, verapamil and SB-710411 0.5, 1.0 and 2.0 μg/kg groups. The rat in the sham group or the control group was injected 0.9% saline solution daily from caudal vein for 3 days at a dose of 5 ml/kg, the rat in verapamil group was intravenously injected (i.v.) daily for 3 days at a dose of 1.6 mg/kg, and the rat in the SB-710411 0.5, 1.0 and 2.0 μg/kg groups was daily administration by venous injection for 3 days at a dose of 0.5, 1.0 and 2.0 μg/kg, respectively.

### Surgical Procedures

The surgical protocol was carried out in according with the methods described by Matsubara et al [23], with some modifications at 1 h after the last drug treatment. Briefly, Sprague-Dawley rats were anaesthetized by intraperitoneal injection of 10% chloral hydrate (350 mg/kg), and maintained by bolus injections of 10% chloral hydrate (60 ~ 80 mg/kg, i.v.) during anaesthesia as required. The neck was dissected and a tracheostomy was performed to provide artificial ventilation (60 strokes/min at a tidal volume of 10 ml/kg). The fourth and fifth ribs on the left side of the chest were cut to perform the thoracotomy and incise the pericardium. The hearts were gently exteriorized and a 5/0 silk suture was passed around the left anterior descending (LAD) coronary artery. The suture then was ligated and the ends of this ligature were passed through a small vinyl tube to form a snare. After 30 min of ischemia, the snare was removed gently and myocardium was reperfused for 90 min. Ischemia was confirmed by ST-segment elevation in the electrocardiogram and color changes in the ischemic myocardial area. Rats in the sham group underwent thoracotomy but the LAD was not ligated. After the I/R protocol, the animals were sacrificed with an overdose of 10% chloral hydrate (500 mg/kg, i.v.), blood samples were collected from the carotid artery, and hearts were harvested for the measurement of infarct size or examinations of histopathology and some related signal molecules expression.

### Measurement of hemodynamics

Rat hemodynamics parameters were continuously monitored before and during cardiac I/R procedure. Briefly, the right common carotid artery was isolated, and a polyethylene tube was placed inside the carotid artery and inserted into the left ventricle. The date of heart rate (HR), mean arterial blood pressure (MABP), left ventricular systolic pressure (LVSP), left ventricular end-diastolic pressure (LVEDP), maximal rate of pressure development for contraction (+dp/dt_max_) and maximal rate of pressure development for relaxation (-dp/dt_max_) were recorded [24].

### Measurement of infarct size

After 90 min of reperfusion, the rat LAD was reoccluded (except the sham group) and 0.5% Evans Blue was injected into the aortic cannula. The hearts were then harvested and were frozen at -20°C in a freezer. The left ventricles were cut into five 2 mm transverse slices and incubated in 1% TTC in phosphate buffer (pH 7.4, 37°C) for 20 min. The normal myocardium was stained blue, the ischemic myocardium was stained light red, and the infracted myocardium did not stain and appeared pale white [25]. Photographing subsequently and measuring the slices to delineate the area of infarct size (IS, TTC-negative) and area at risk (AAR, TTC-stained). Myocardial infarct sizes (IS/AAR%) were calculated by computerized planimetry Useing ImageJ version 1.6 (National Institutes of Health, Bethesda, Md, USA) [26].

### Measurement of activities of CK-MB, LDH and cTnI

Above blood samples were collected and centrifugated at 3000 g at 4°C for 10 min. The activities of CK-MB, LDH in the serum were detected with the commercially available assay kit according to the procedures. The serum cTnI level was detected by using a rat ELISA kit.

### Histopathological examination

The harvested heart was fixed in 10% formalin solution, dehydrated, embedded in paraffin, and cut into 4 μm sections using a microtome. The sections were stained with hematoxylin and eosin (H&E) and using a light microscope to examine the architecture of the myocardium.

### Western blotting

The total proteins in the left ventricular tissues of rat hearts were extracted with the method previously described [27]. Briefly, the tissue was homogenized and lsyed with RIPA lysis buffer (1 mM sodium orthovanadate, 50 mM Tris HCl, 1%Triton X-100,150 mM NaCl, 1 mM bglycerophosphate, 1 mM DTT and protease inhibitor) on ice for 30 min and then centrifμgated at 12,000 g for 10 min at 4°C. The protein in the supernatants was determined spectrophotometrically at a 562 nm wavelength using bicinchoninic acid (BCA) protein assay kit. The total protein (30μg) was separated by 10% SDS-polyacrylamide gel electrophoresis (PAGE) and then transferred to a polyvinylidene difluoride (PVDF) membranes. The membranes were blocked with buffer containing 5% skim milk in TBS-T containing 10 mmol/L Tris–HCl (pH 6.8), 150 mmol/L NaCl, and 0.05% Tween 20 followed by incubation overnight at 4°Cwith the primary antibodies of UT or ROCK_1_ or ROCK_2_. The membranes were washed three times with 0.1% Tween-20 for 15 minutes and subsequently incubated with appropriate secondary antibody for 1 h at 37°C. Relative intensity of the band was determined by densitometry of radioautograph films.

### RhoA activity

RhoA activity in rat myocardial tissue was determined by using absorbance based G-LISA RhoA activation assay Biochem kitTM. Frozen myocardial tissues were homogenized in lysis buffer and the protein concentration was determined according to the manufacturer’s instructions. RhoA activity is detected by measuring absorbance at 490 nm using a microplate spectrophotometer after indirect immunodetecrtion [28].

### Statistical analysis

All numerical data are presented as means ± SD. By using SPSS version 10.0 software, one-way analysis of variance or non-parametric test was applied for assessing statistically significant differences among various groups. Value of *p* < 0.05 was considered statistically significant.

## Results

### Effect of SB-710411 on hemodynamic parameters of rats subjected to myocardial I/R injury

As shown in Table 1, SB-710411 did not affect the hemodynamic parameters in normal rat as indicated by no significant difference in baseline values of HR, MABP, LVSP, LVEDP, +dp/dt_max_ and–dp/dt_max_ among various groups. Table 1 also shows that myocardial I/R injury induced prominent decreases in HR, MABP, LVSP, +dp/dt_max_ and -dp/dt_max_ and increase in LVEDP compared with those in the sham group (*p <* 0.05 or *p <* 0.01). However, treatment with SB-710411 1.0 and 2.0 μg /kg markedly inhibited these changes of hemodynamic parameters (*p* < 0.05) except for LVSP.

**Table 1:** Effect of SB-710411 on hemodynamic parameters of rats subjected to the left anterior descending artery occlusion and reperfusion.

Group	Dose (μg /kg)	Parameters	Baseline	Ischemia 30 min	Reperfusion		
					30 min	60 min	90 min
Sham	—	HR(beats/min)	424.3±50.6	425.4±47.9	412.7±45.8	416.8±47.9	420.8±52.0
Control	—	HR(beats/min)	441.6±50.7	438.9±42.4	430.6±50.6	416.5±48.6	389.8±46.3*
SB-710411	1.0	HR(beats/min)	428.4±56.3	430.6±51.3	426.8±49.0	420.4±53.9	413.9±52.6
	2.0	HR(beats/min)	437.0±49.2	433.6±48.7	428.6±50.3	424.0±57.9	415.8±50.3
Sham	—	MABP(mmHg)	121.4±11.4	120.9±10.7	118.8±10.5	116.6±9.8	119.9±9.3
Control	—	MABP(mmHg)	123.6±12.9	86.7±10.8**	89.8±11.1**	82.8±10.1**	80.9±9.9**
SB-710411	1.0	MABP(mmHg)	119.2±11.7	90.6±11.2	93.9±8.7	88.7±8.7	86.1±8.0
	2.0	MABP(mmHg)	124.1±9.4	92.6±10.5	94.8±7.0	89.2±7.7	84.3±8.1
Sham	—	LVSP(mmHg)	103.1±9.0	102.1±8.2	103.9±9.7	101.7±9.3	100.8±10.7
Control	—	LVSP(mmHg)	106.5±10.6	90.6±13.8*	89.0±11.2*	87.9±10.5*	84.9±9.1*
SB-710411	1.0	LVSP(mmHg)	100.5±12.0	90.8±10.3	92.5±9.4	90.6±10.8	87.4±11.8
	2.0	LVSP(mmHg)	102.5±10.7	93.5±10.0	94.7±11.3	91.1±11.8	86.0±10.7
Sham	—	LVEDP(mmHg)	7.89±2.33	7.8±2.2	7.9±2.6	8.2±2.2	8.1±2.2
Control	—	LVEDP(mmHg)	8.06±1.89	17.2±3.9**	19.0±3.9**	19.3±3.8**	19.9±3.8**
SB-710411	1.0	LVEDP(mmHg)	8.02±2.05	13.9±4.0^#^	14.2±3.8^#^	14.9±3.3^#^	15.8±3.3^#^
	2.0	LVEDP(mmHg)	8.17±1.92	13.0±3.4^#^	13.9±3.2^#^	14.2±3.6^#^	15.2±3.5^#^
Sham	—	+dp/dt_max_ (1000mmHg/S)	3.9±0.3	3.9±0.3	4.0±0.3	3.9±0.3	4.0±0.3
Control	—	+dp/dt_max_ (1000mmHg/S)	3.9±0.3	2.3±0.3**	2.3±0.3**	2.3±0.3**	2.2±0.3**
SB-710411	1.0	+dp/dt_max_ (1000mmHg/S)	34.0±0.3	2.7±0.3^#^	2.7±0.3^#^	2.6±0.3^#^	2.6±0.3^#^
	2.0	+dp/dt_max_ (1000mmHg/S)	3.9±0.3	2.7±0.3^#^	2.7±0.3^#^	2.6±0.3^#^	2.6±0.3^#^
Sham	—	-dp/dt_max_ (1000 mmHg/S)	3.8±0.3	3.8±0.3	3.8±0.3	3.8±0.3	3.8±0.3
Control	—	-dp/dt_max_ (1000 mmHg/S)	3.8±0.3	2.1±0.3**	2.2±0.3**	2.1±0.3**	2.1±0.3**
SB-710411	1.0	-dp/dt_max_ (1000 mmHg/S)	3.8±0.3	2.5±0.3^#^	2.5±0.3^#^	2.5±0.3^#^	2.4±0.3^#^
	2.0	-dp/dt_max_ (1000 mmHg/S)	3.8±0.3	2.5±0.3^#^	2.5±0.3^#^	2.5±0.3^#^	2.5±0.3^#^

Results are expressed as the mean ± SD (n = 8 per group).

*p < 0.05

**p < 0.01 vs. the sham group

^#^*p <* 0.05 vs. the control group.

### Effect of SB-710411 on I/R injury-induced myocardial infarction

**Fig 1:**
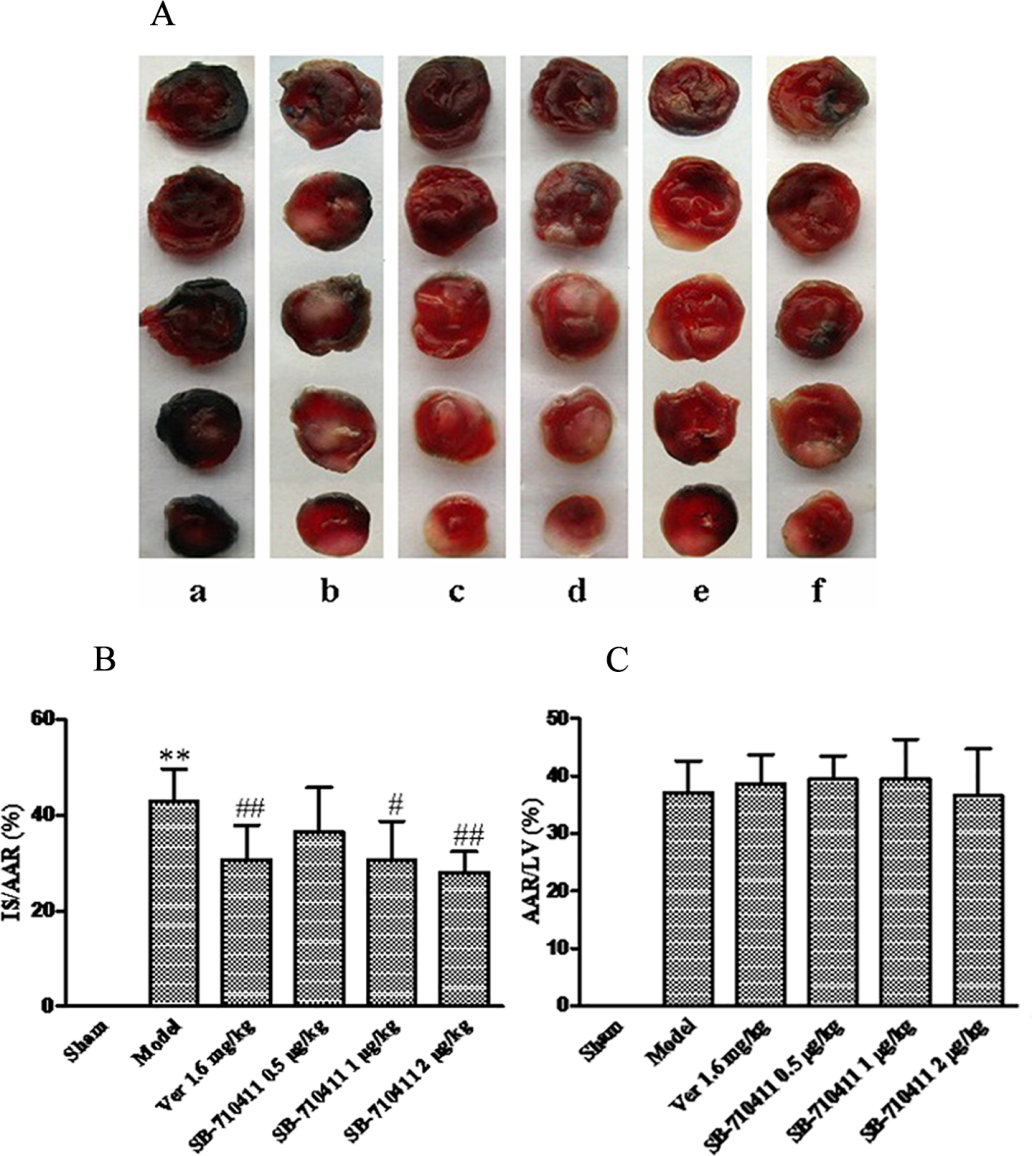
Effects of SB-710411 on ischemia/reperfusion injury-induced myocardial infarction in rats. Evans Blue and 2,3,5-triphenyltetrazolium chloride (TTC) double staining was used to determine the ischemic size and infarction size. **A.** Rat myocardial slice stained by Evans blue and TTC. The normal area was stained blue, the area at risk (AAR) was stained red, and the area of infract size (IS) was stained pale white. **a:** Sham; **b:** Control; **c:** 1.6 mg/kg verapamil; **d ~ f:** 0.5, 1.0 and 2.0 μg/kg SB-710411. **B.** Effect of SB-710411 on the myocardial infarction. The IS was normalized by expressing it as a percentage of the AAR (IS/AAR). **C.** Effect of SB-710411 on the AAR. AAR was expressed as a percentage of the left ventricular area (AAR/LV). Results are expressed as mean ± SD (n = 8 per group). **p < 0.01 vs. the sham group; ^#^*p <* 0.05, ^##^*p <* 0.01 vs. the control group.

As shown in Fig 1, no area at risk (AAR) and infarct size (IS) was observed in the sham group. I/R injury induced significant myocardial infarct in the control group by the measurement of the IS/AAR ratio (42.83% ± 6.79%). However, the IS/AAR ratio was profoundly reduced in the SB-710411.0 and 2.0 μg/kg groups (30.69% ± 8.05% and 28.01% ± 4.32%) compared with that in the control group (*p <* 0.05 or *p <* 0.01). While no significantly statistical difference for AAR was observed among groups except for the sham group. Verapamil 1.6 mg/kg had a similar effect in reducing the IS/AAR ratio (*p* < 0.01).

### Effect of SB-710411 on ST-segment change after myocardial I/R injury

**Fig 2:**
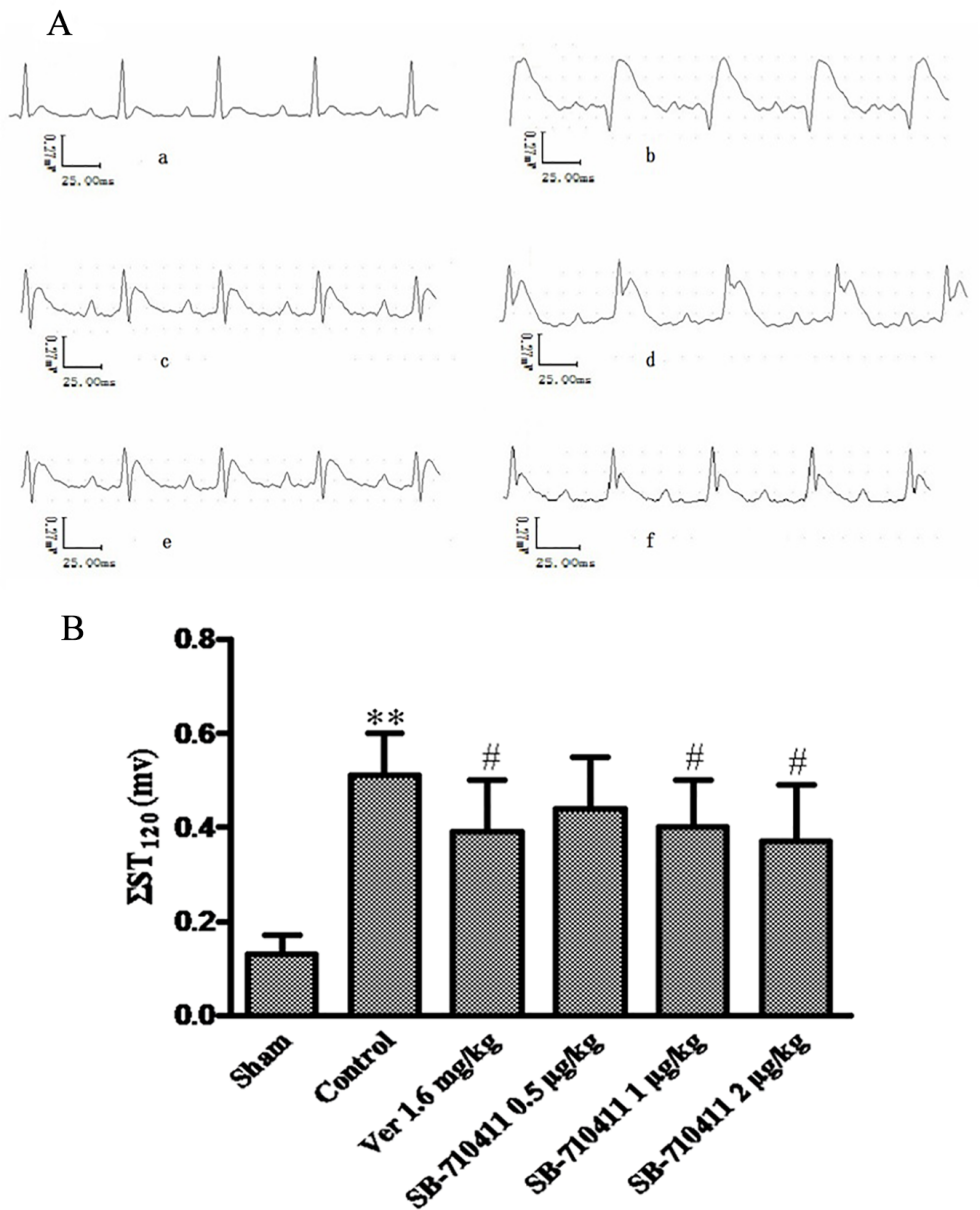
Effect of SB-710411 on ST-segment elevation after myocardial ischemia/reperfusion in rats. Rats were subjected to 30 min of myocardial ischemia followed 90 min of reperfusion, and electrocardiogram (ECG) was measured. **A.** The traces of the ECG in various groups. **a:** Sham; **b:** Control; **c:** 1.6 mg/kg verapamil; **d ~ f:** 0.5, 1.0 and 2.0 μg/kg SB-710411. **B.** Effect of *S*B-710411 on ST-segment elevation. Results are expressed as mean ± SD (n = 8 per group). **p < 0.01 vs. the sham group; ^#^*p <* 0.05, ^##^*p <* 0.01 vs. the control group.

As shown in Fig 2, I/R injury caused severe ST-segment (ΣST) elevation in the control group. However, Treatment with SB-710411 could significantly attenuate the increase of ΣST (*p* < 0.05). Verapamil had a similar effect in attenuating the increase of ΣST (*p* < 0.05).

### Effect of SB-710411 on myocardial I/R injury-induced increases of serum CK-MB and LDH

**Fig 3:**
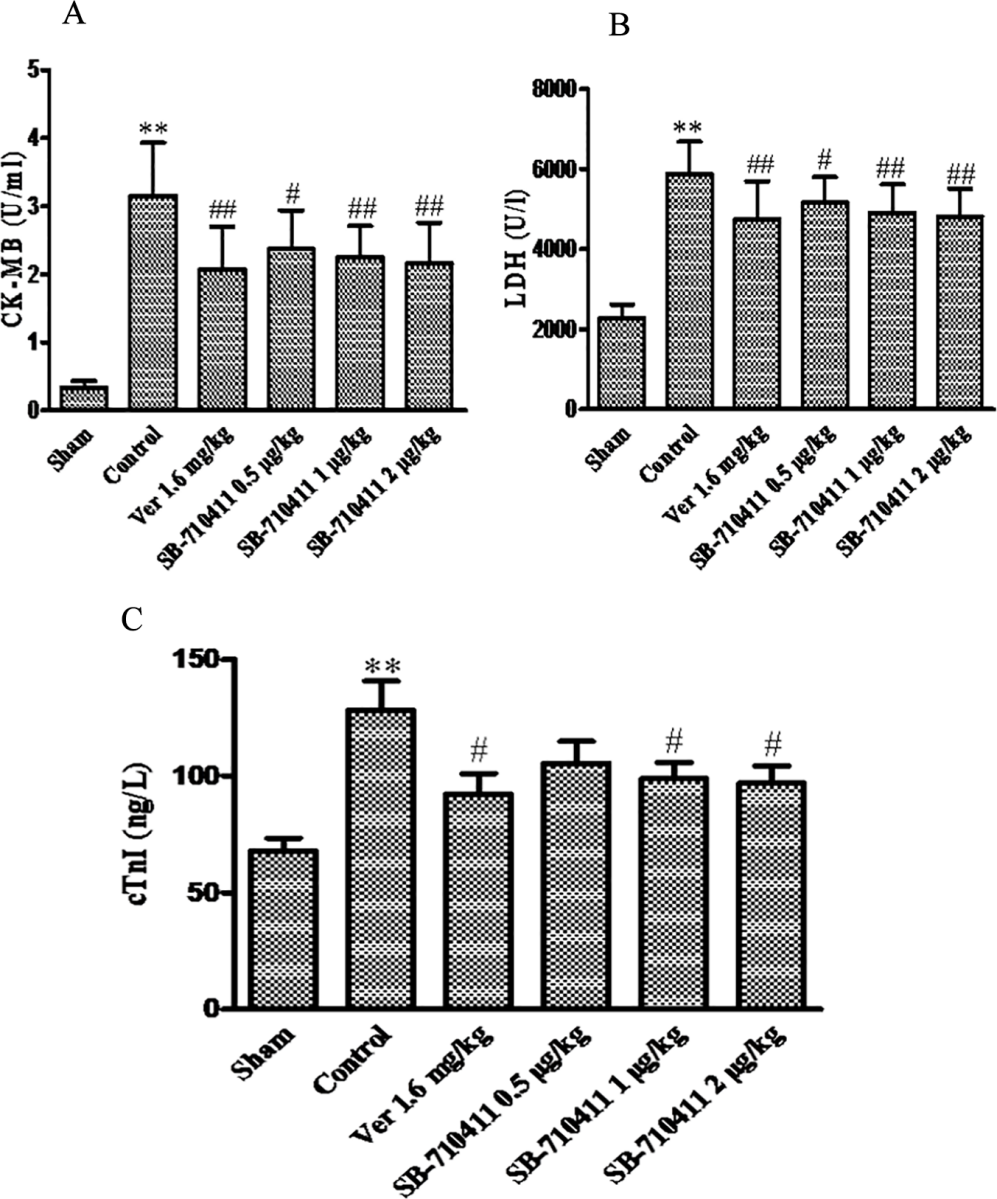
Effect of SB-710411 on the serum creatine phosphokinase-MB (CK-MB) and lactate dehydrogenase (LDH) activities and cardiac troponin I (cTnI) level in rats subjected to 30 min of ischemia followed 90 min of reperfusion. A. CK-MB activity. B. LDH activity. **C.** cTnI level. Results are expressed as mean ± SD (n = 8 per group). **p < 0.01 vs. the sham group; ^#^*p <* 0.05, ^##^*p <* 0.01 vs. the control group.

As shown in Fig 3, the activities of serum CK-MB and LDH in the control group have significantly increased compared with those in the sham group (*p* < 0.01). Treatment with SB-710411 could significantly inhibit the increases of CK-MB and LDH activities. CK-MB activity was reduced from 3.2 ± 0.8 U/mL in the control group to 2.4 ± 0.6 U/mL, 2.2 ± 0.5 U/mL and 2.1 ± 0.6 U/mL in 0.5, 1.0, and 2.0 μg/kg SB-710411 groups, respectively (*p* < 0.05 or *p* < 0.01). Similarly, 0.5, 1.0, and 2.0 μg/kg SB-710411 markedly decreased LDH activity from 5884.0 ± 799.4 U/L in the control group to 5168.1 ± 637.1 U/L, 4909.3 ± 7707.1 U/L and 4815.4 ± 697.0 U/L, respectively (*p* < 0.05 or *p* < 0.01). Verapamil had a similar effect in decreasing the CK-MB and LDH activities (*p* < 0.01).

### Effect of SB-710411 on myocardial I/R injury-induced increase of serum cTnI

Fig 3 also shows that the serum cTnI level in the control group (128.1 ± 35.7 ng/L) was significantly increased compared with that in the sham group (67.9 ± 15.8 ng/L). Treatment with SB-710411 1.0 and 2.0 μg/kg could significantly inhibit this increase, the cTnI level was reduced to 98.8 ± 20.1 ng/L and 96.9 ± 21.2 ng/L, respectively (*p* <0.05). Verapamil also markedly decreased the serum cTnI level (*p* < 0.05).

### Effect of SB-710411 on the histopathological alteration in rat myocardium

**Fig 4:**
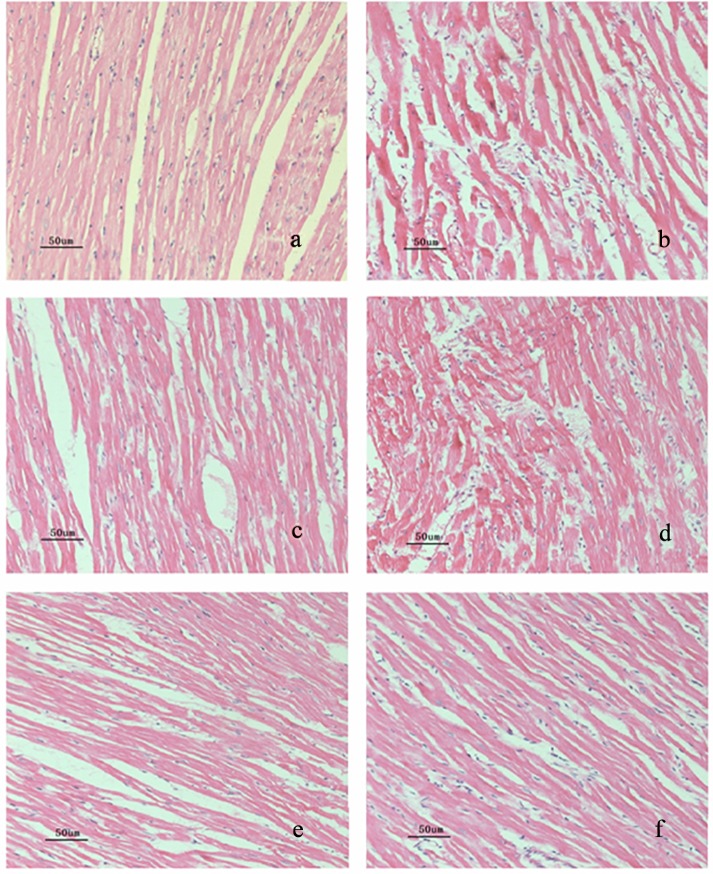
Ischemia/reperfusion-induced the histopathological alteration in rat myocardium (hematoxylin and eosin staining, magnification 200×). The black bar represents 50 μm. Histological analysis showed that there is normal intact architecture of the myocardium in the sham group, while an irregular arrangement, obvious necrosis, marked edema, neutrophilic infiltration, mild inflammation, myofibril loss and cracks were observed in the control group. 1.0 and 2.0 μg/kg SB-710411 and verapamil 1.6 mg/kg significantly alleviated the myocardial damage. **a**: Sham; **b**: Control; **c**: 1.6 mg/kg verapamil; **d ~ f**: 0.5, 1.0 and 2.0 μg/kg B-710411.

To evaluate the extent of cardiac lesion after I/R, rat myocardial slices were stained with H&E. As shown in Fig 4 and Table 2, the histopathological examination revealed that in the sham group, non-infarcted myocardium was characterized by organized pattern and normal architecture, cardiomyocytes presented normal size and clear boundaries. Whereas the myocardium in the control group occurred obvious necrosis, extensive edema, neutrophilic infiltration, and mild inflammation, cardiomyocytes arranged irregularly and intercellular space enlarged. In some area, there were myofibril loss and cracks. Treatment with SB-710411 significantly relieved these myocardial lesions. Cardiomyocytes in SB-710411 1.0 and 2.0 μg/kg groups ranked in order, the necrosis, inflammatory cells infiltration and edema in the myocardium reduced remarkably. The myocardial histologic scores were significantly decreased by administration with SB-710411 compared with those in the control group. Administration of verapamil had a similar effect as that observed in the SB-710411 treated groups.

**Table 2:** Effect of SB-710411 on myocardial histologic scores of rat subjected to the left anterior descending artery occlusion and reperfusion.

Group	Dose (/kg)	n	Myocardial necrosis					Interstitial edema					Infiltration of inflammatory cells				
			–	+	++	+++		–	+	++	+++		–	+	++	+++	
Sham	/	8	8	0	0	0		8	0	0	0		8	0	0	0	
Model	/	8	0	0	4	4	**	0	0	3	5	**	0	0	4	4	**
Verapamil	1.6 mg	8	0	6	2	0	^#^	0	7	1	0	^#^	0	6	1	1	^#^
SB-710411	0.5 μg	8	0	4	2	2		0	1	4	3		0	2	3	3	
	1.0 μg	8	0	5	2	1	^#^	0	5	3	0	^#^	0	5	3	0	^#^
	2.0 μg	8	0	6	2	0	^#^	0	6	2	0	^#^	0	6	1	1	^#^

Score

–, no abnormal findings

**+**, mild

**++**, moderate

**+++**, severe.

* *p <* 0.05

**p < 0.01 vs. the sham group

^#^*p <* 0.05 vs. the control group.

### Effect of SB-710411 on expression of UTR protein

**Fig 5:**
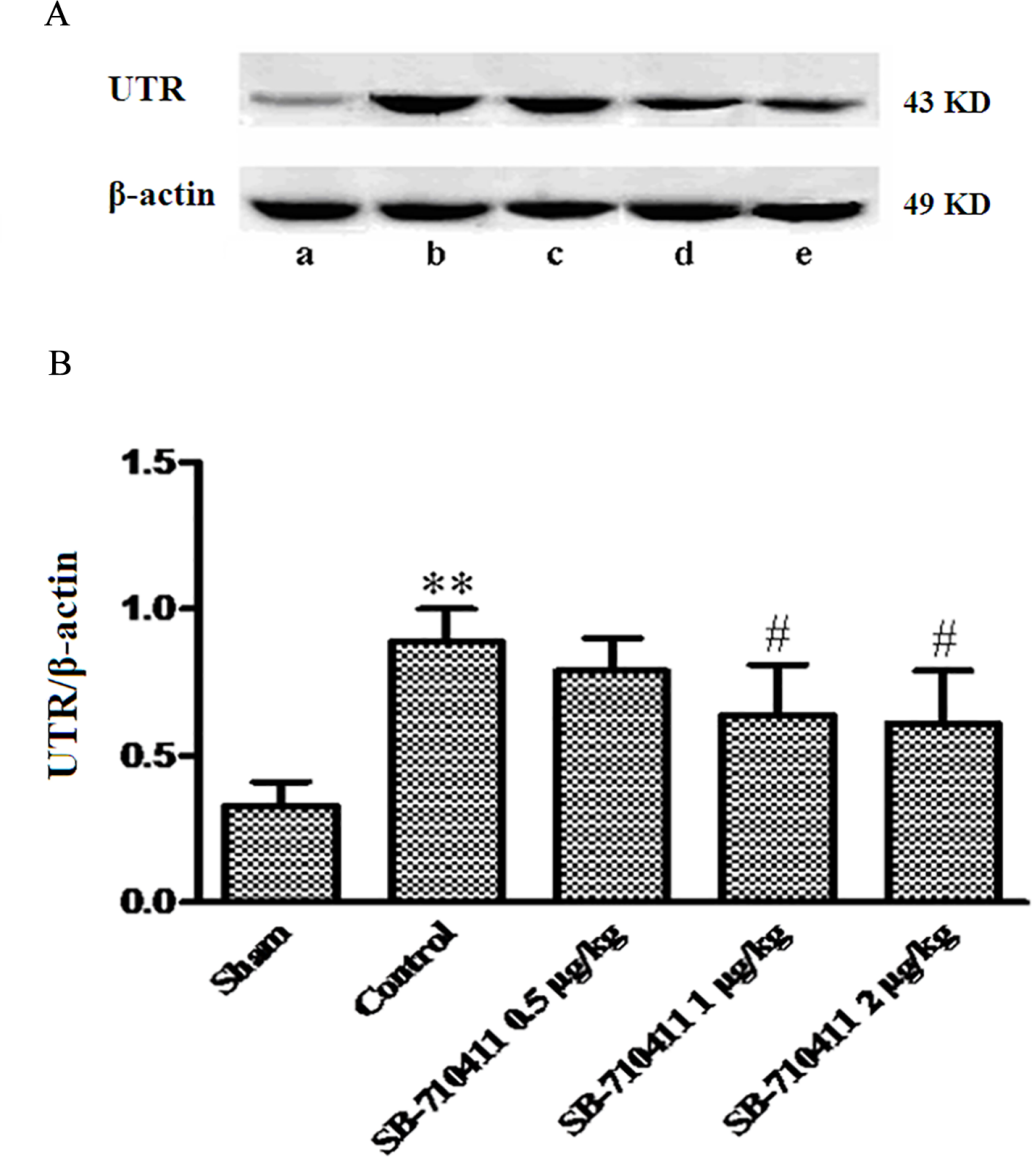
Effect of SB-710411 on the expression of UTR protein in rat myocardium after ischemia/reperfusion. **A.** UTR protein was detected by Western blot method. β-actin was used as the loading control. **B**. Densitometric quantification of UTR protein expression. **a:** Sham; **b:** Control; **c ~ e:** 0.5, 1.0 and 2.0 μg/kg SB-710411. Results are expressed as mean ± SD (n = 4 per group). **p < 0.01 vs. the sham group; ^#^*p <* 0.05, ^##^*p <* 0.01 vs. the control group.

As shown in Fig 5, the expression of UTR protein in rat myocardium was detected by using Western bolt method in each group. In comparison with the sham group, the expression of UTR protein was dramatically up-regulated in the control group (*p* < 0.01). By contrast, SB-710411 could significantly inhibit myocardial I/R injury-induced up-regulation of UTR expression compared with that in the control group (*p* < 0.05).

### Effect of SB-710411 on RhoA activity

**Fig 6:**
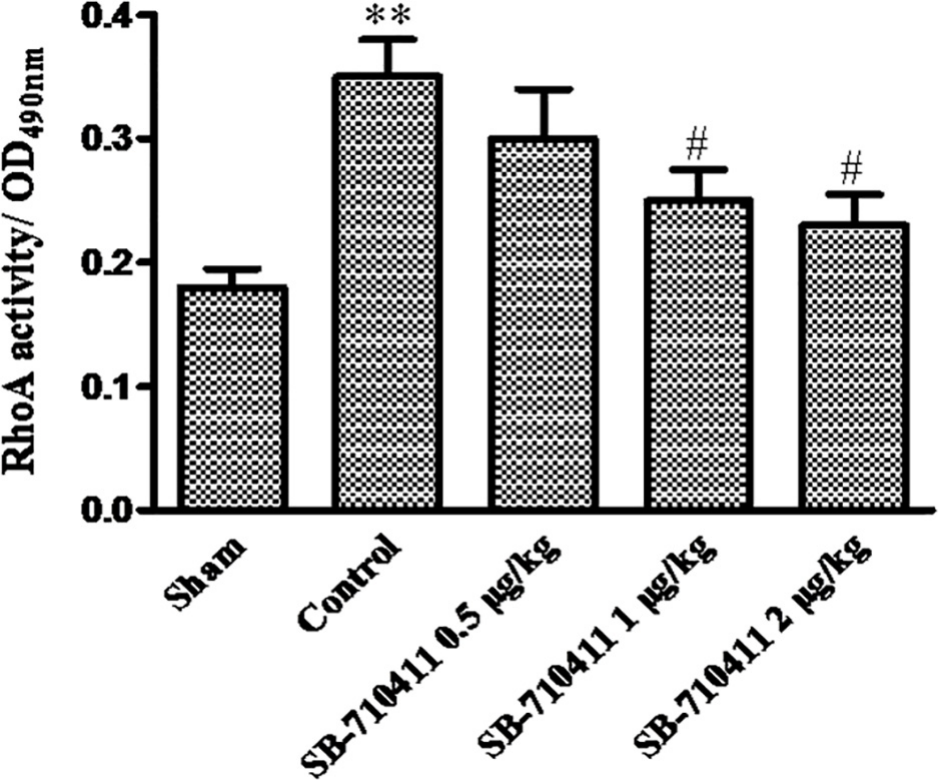
Effect of SB-710411 on RhoA activity in rat myocardium after ischemia/reperfusion. The activity of RhoA in myocardium was detected by luminescence-based G-LISA™ assay. Results are expressed as mean ± SD (n = 4 per group). **p < 0.01 vs. the sham group; ^#^*p <* 0.05, ^##^*p <* 0.01 vs. the control group.

In order to study the role of RhoA/ROCK pathway in the protective effect of SB-710411, RhoA activity was detected in the myocardium. As shown in Fig 6, RhoA activity in myocardium of the control group (0.35 ± 0.06) increased significantly compared with that in the sham group (0.18 ± 0.03). Treatment with SB-710411 1.0 and 2.0 μg/kg could evidently inhibit the increases of RhoA activity. The activity was reduced to 0.25 ± 0.05 and 0.23 ± 0.05, respectively (*p* < 0.05).

### Effect of SB-710411 on expressions of ROCK_1_ and ROCK_2_ proteins

**Fig 7:**
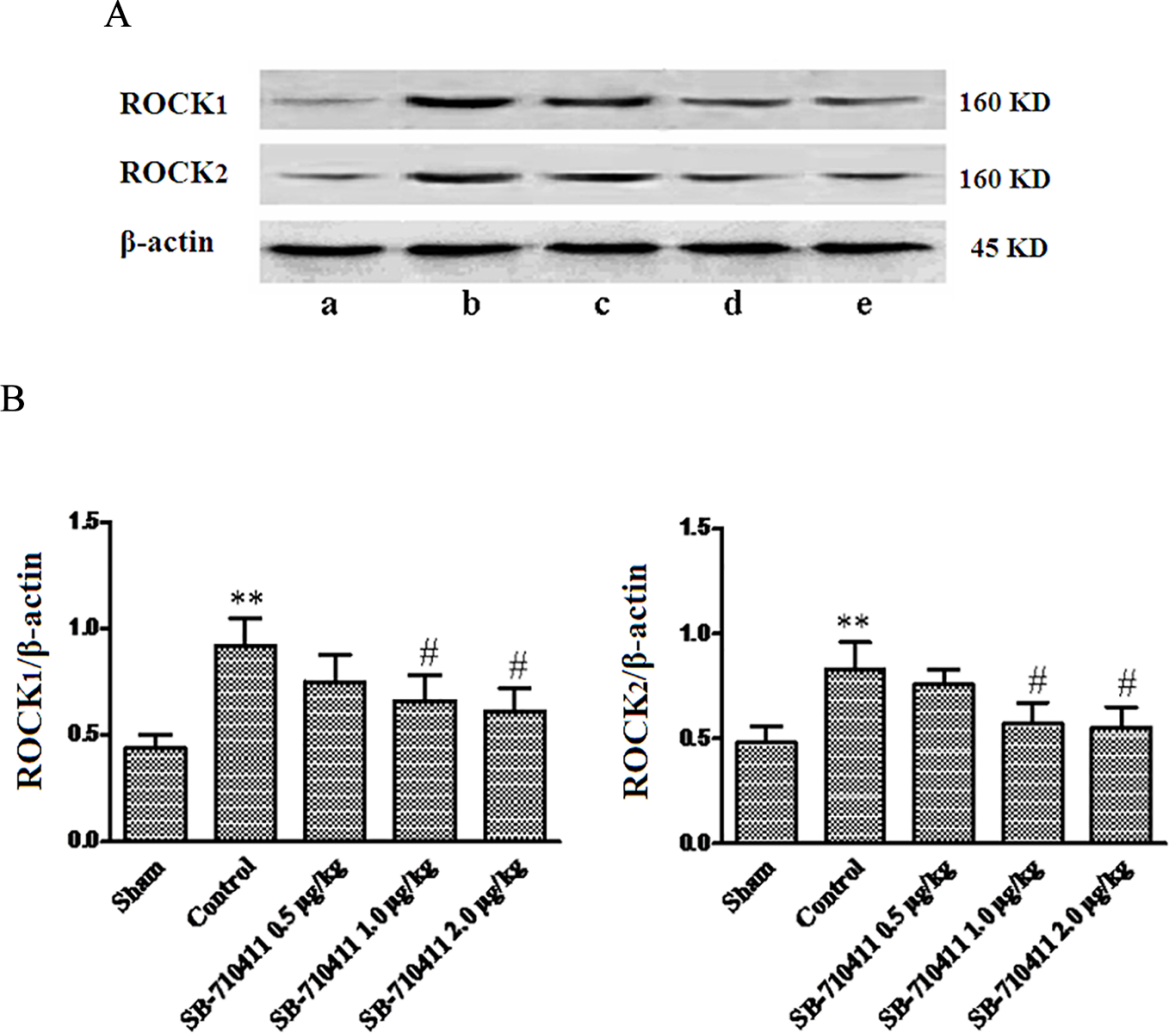
Effect of SB-710411 on protein expressions of ROCK_1_ and ROCK_2_ in rat myocardium injured by ischemia/reperfusion. **A.** ROCK_1_ and ROCK_2_ proteins were detected by Western blot method. β-actin was used as the loading control. **B.** Densitometric quantification of ROCK_1_and ROCK_2_ protein expression. **a:** Sham; **b:** Control; **c ~ e:** 0.5, 1.0 and 2.0 μg/kg SB-710411. Results are expressed as mean ± SD (n = 4 per group). **p < 0.01 vs. the sham group; ^#^*p <* 0.05, ^##^*p <* 0.01 vs. the control group.

As shown in Fig 7, the protein expressions of ROCK_1_ and ROCK_2_ in rat myocardium were detected in the sham group (0.44 ± 0.06 and 0.48 ± 0.08). Myocardial I/R injury dramatically caused increases of both ROCK_1_ and ROCK_2_ protein expressions (0.92 ± 0.13 and 0.83 ± 0.13) (*p* < 0.01). SB-710411 1.0 and 2.0 μg/kg could significantly inhibit the increases of ROCK_1_ and ROCK_2_ protein expressions (0.66 ± 0.12 and 0.61 ± 0.11; 0.57 ± 0.10 and 0.55 ± 0.10) compared with those in the control group (*p <* 0.05).

## Discussion

The urotensinergic system plays a crucial physiological and pathophysiological role in cardiovascular homeostasis. A lot of evidence has shown that U-II/UTR system is up-regulated in cardiovascular disorders, such as atherosclerosis, heart failure, hypertension and ischemic heart disease [29–30]. Despite several reports documenting increased UTR expression in the ventricular myocardium from congestive heart failure rat [31–32], the change of UTR expression in cardiac I/R injury is unclear. Our result demonstrated that cardiac I/R injury markedly up-regulated the UTR protein expression in rat myocardium. The result suggests that UTR may have a pathological role in myocardial I/R injury, and the antagonism on UTR may be a novel therapeutic target.

UTR antagonists have been developed, and their effects were studied in animal models of cardiovascular disease point to promising therapies for the future. SB-710411 is a selective UTR antagonist in rat. It could inhibit the vasoconstrictor response of rat aorta to human U-II in a competitive manner [17] and modulate collagen synthesis and accumulation in rat aortic vascular smooth muscle cells through reducing the expression of Smad_2_ and Smad_3_ [18]. However, SB-710411 was reported to having an intrinsic activity in monkey arteries acting as an efficacious vasoconstrictor [33]. A previous study demonstrates that GSK1562590, another selective UTR antagonist exhibits an insurmountable antagonism at UTR in contraction of arteries from rat and human UTR transgenic mouse arteries, but a competitive antagonistic effect in monkey arteries. Dissociation of GSK1562590 binding to UTR was profoundly slower at rat than monkey UTR [34]. The results suggest that effect of UTR antagonist may be variable according to the UTR in different species, and perhaps the characteristics of human UTR is similar to that of rat UTR. Hence, it is possible that SB-710411 has relaxation in human artery as it does in the rat blood vessel. The present study also demonstrates that SB-710411 significantly decreases myocardial I/R injury-increased the UTR protein expression in rat myocardium, and has a significant protective effect on the cardiac I/R injury. This cardioprotection was indicated by improvement of hemodynamics, lower serum CK-MB and LDH activities, attenuation of the increase of ΣST in ECG, reduced infarct size and amelioration of the histopathological alteration in the myocardium.

CTnI is a protein with high sensitivity and specificity for myocardial damage and considered a special biomarker for clinical detection of cardiac cellular injury [35]. In the present study, rat serum cTnI level increased significantly after myocardial I/R injury, but treatment with SB-710411 remarkably reduced the increase of cTnI level. This result is in accord with the aforementioned protective effect of SB-710411 against myocardial I/R injury in rat. In addition, the protective effect of SB-710411 on rat myocardial I/R injury is agreement with our previous study that urantide, another UTR antagonist, has a potent protective effect against myocardial I/R injury [10].

Once bound to its receptor, human U-II leads activation of different signaling pathway systems such as p38MAPK, ERK1/2 and PI3K-Akt [10, 36]. RhoA/ROCK is also an important intracellular signaling transduction pathway, which is associated with diverse cellular functions. RhoA is one of small guanosine-5′-triphosphate-binding protein and exhibits GDP/GTP-binding activity. RhoA is active in GTP-bound state and inactive in GDP-bound state. ROCK is the major downstream effector of RhoA. RhoA could directly interact with ROCK and play important roles in many cardiovascular pathogenesis including arterial hypertension, atherosclerosis, heart attack, vascular remodeling, myocardial hypertrophy, and myocardial I/R injury [37–38]. Increasing evidences demonstrate that activities of human U-II such as muscle contraction, actin cytoskeleton organization and proliferation of vascular smooth muscle cells are mediated by activation of the RhoA/ROCK pathway [39].

ROCK has two isoforms, ROCK_1_ and ROCK_2_ [40], also known as ROCKα and ROCKβ. ROCK_1_ and ROCK_2_ have similar amino acid (65% identity) and kinase domains (92% identity). They contain a C-terminal pleckstrin-homology domain and a catalytic kinase domain at the N-terminus. Both ROCK_1_ and ROCK_2_ are expressed in heart and involved in myocardial I/R injury. It was found that coptisine, an isoquinoline alkaloid extracted from *Chinese goldthread* (Coptis chinensis), protects rat heart against myocardial I/R injury by inhibiting ROCK_1_/ROCK_2_ expression [21]. In the present study, myocardial I/R injury notably induced increases of the RhoA activity and the expressions of ROCK_1_ and ROCK_2_ protein, but the treatment of SB-710411 could significantly inhibit the increases of RhoA activity and the expressions of ROCK_1_ and ROCK_2_. The results demonstrate that activation of RhoA/ROCK pathway occurs during cardiac I/R process, and the suppression of RhoA/ROCK signaling pathway is involved in the cardioprotection of SB-710411 in rats subjected to myocardial I/R injury.

Histopathological examination revealed that administration of SB-710411 significantly alleviated I/R injury-induced myocardial structural changes. In consideration of the role of RhoA/ROCK pathway in myocardial I/R injury, this alleviation of myocardial structure at least partially contributed to the aforementioned inhibition of SB-710411 on RhoA/ROCK pathway.

In conclusion, this study provides novel evidence that cardiac I/R injury increases myocardial UTR expression, and UTR antagonists SB710411exerts protective effect against myocardial I/R injury in rats. The cardioprotective effect of SB710411 may be associated with antagonism of UTR, and subsequent inhibition of the RhoA-ROCK signaling pathway.
